# The effect of gamma irradiation on the structural properties of olivine

**DOI:** 10.1007/s10967-018-5849-6

**Published:** 2018-04-06

**Authors:** Mariola Kądziołka-Gaweł, Mateusz Dulski, Lech Kalinowski, Marcin Wojtyniak

**Affiliations:** 1 Institute of Physics, University of Silesia in Katowice, 75 Pulku Piechoty 1a, 41-500 Chorzow, Poland; 2 Institute of Material Science, University of Silesia in Katowice, 75 Pulku Piechoty 1a, 41-500 Chorzow, Poland; 3 Silesian Center for Education and Interdisciplinary Research, 75 Pulku Piechoty 1a, 41-500 Chorzow, Poland

**Keywords:** Olivine, Gamma irradiation, X-ray diffraction, Raman spectroscopy, Mössbauer spectroscopy

## Abstract

Gamma irradiation studies of (Mg_0.905_Fe_0.095_)_2_SiO_4_ olivine were performed using X-ray fluorescence method, X-ray diffraction, Raman and Mössbauer spectroscopy. The absorbed doses were 300, 600 and 1000 Gy. Small irradiation doses cause an increase of lattice vibrations and small deformation of both M1 and M2 octahedron. The observed effect is similar to the results expose to high temperature. However, the small deformation takes place only in unit cell of Olivine’s structure.

## Introduction

**Fig. 1:**
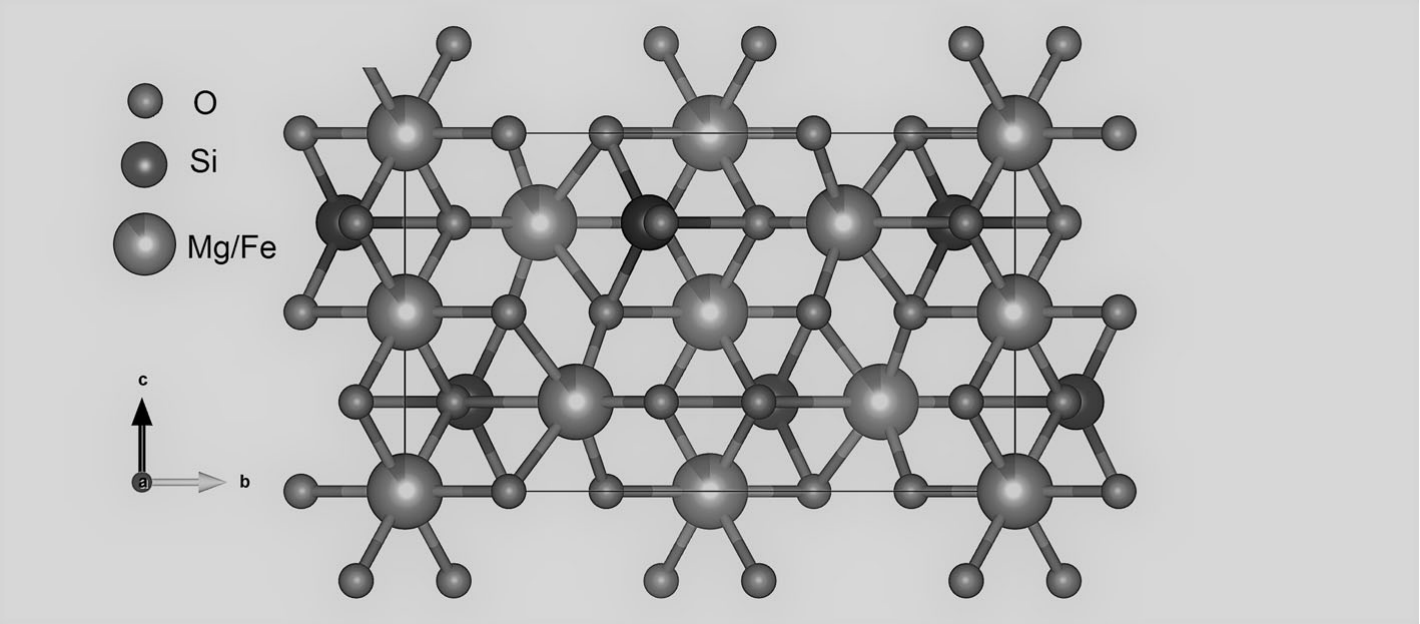
Unit cell of olivine structure viewed along [100]

Olivine is one of the simplest silicate minerals that can be found in igneous rocks on our planet. Olivine (Fe,Mg)_2_SiO_4_ is orthosilicate with an orthorhombic crystal structure that is characterized by a distorted hexagonal close packing of oxygen ions with Si on tetrahedral interstices and Mg and Fe ions on octahedral sites (Fig. 1). The former is a regular octahedron, labeled M2, and the latter is a distorted octahedron, labeled M1. The Fe(II) and Mg(II) have no particular preference for either site. Although extensive studies have been conducted on olivine over a range of compositions, temperatures and pressures [1–3] using a variety of methods, however, more studies are still need on several aspects of the nature of olivine and its variations to create resolution over those aspects.

With the development of radiation physics, the study of the action of nuclear radiation on the structural properties and phase transitions in solids began [4]. For instance, the irradiance of drugs, or other medicinal products, by suitable dose of an ionizing radiation in suitable einveironment, couse sterylity condition [5, 6]. Radiation (gamma radiation, X-rays, electron beam) can induce numerous changes in the physical properties of minerals, one of the most obvious effects being visible coloration, which is caused by the color centers (defects). Also, irradiation influences the formation and the alteration of the structure and morphology of the crystals [7]. Studies on different kinds of glasses and crystal silicate demonstrated their potential application for radiation dosimetry or addressed their crystalographical aspects [8, 9].

This paper presents comparative results of the characterization of olivine structure as a function of gamma irradiation at room temperature using the X-ray fluorescence method, X-ray diffraction, Raman spectroscopy and Mössbauer spectroscopy. The goal of this work was to link a structural study of olivine to a characterization of the electronic configuration of iron as a function of absorbed dose from 300 to 1000 Gy. Moreover, this work tries to give the answer as to how the results using the XRD and Mössbauer methods connected with the structure of the investigated material should be interpreted. It also helps to better understand physical properties of this mineral.

## Experimental

A natural sample of olivine from Lanzarote was chosen to perform a detailed analysis. Lanzarote Island is a result of mantle plume activity beneath the Canary Islands. The pulverized sample of olivine was exposed to electromagnetic ionizing radiation resulting in absorbed dose of 300, 600 and 1000 Gy. Doses were delivered using gamma ray emitted by ^137^Cs isotope with the energy 0.66 MeV. The distance from exposure was 0.5 cm and irradiation time totalled about 2 weeks. The irradiation was conducted in three series 300, 300 and finally 400 Gy; therefore, the total absorbed dose by sample was 1000 Gy. After each step of irradiation XRD, Raman and Mössbauer measurements were done.

The chemical composition was determined using X-ray fluorescence (XRF) from ZSX Primus II Rigaku spectrometer. The spectrometer, equipped with the 4 kW, 60 kV Rh anode and wavelength dispersion detection system, allowed for the analysis of the elements from Be to U. No external standards were necessary, only the internal standards coupled with the fundamental parameters method (theoretical relationship between measured X-ray intensities and the concentrations of elements in the sample) were implemented.

The X-ray diffraction studies (XRD) of the investigated olivine were conducted at room temperature using a Siemens D5000 X-ray diffractometer and Cu:K_α_. Rietveld refinement was performed in a licensed Xpert High Score Plus with PDF-4 crystallography database. Structure visualizations were made using Vesta program [10].

A WITec confocal Raman microscope CRM alpha 300 equipped with a solid-state laser (λ = 532 nm) and a CCD camera was applied to determine the crystal structure of non-treated and gamma-treated olivine (= forsterite) and indirectly applied to determine the chemical composition through the analysis of magnesium/iron oxide linkage and silicate groups. Forsterite grains have been all the time oriented in XX plane due to high-sensitivity to polarization. The excitation laser radiation was coupled into a microscope through a single-mode optical fiber with a 50 mm diameter. An Olympus MPLAN (50×/0.50NA) air objective and monochromator with a 600 line/mm grating were used. Raman scattered light was focused onto a multi-mode fiber (50 mm diameter). All spectra were accumulated by ten scans with integration time of 10 s and the resolution of 3 cm^−1^. The monochromator of the spectrometer was calibrated using the Raman scattering line of a silicon plate (520.7 cm^−1^). The fluorescence and baseline correction, as well as band fitting analysis by Voigt function, were performed using GRAMS software package.

The initial samples were powdered in an agate ball mill and then prepared in a shape of a thin disk absorber. ^57^Fe Mössbauer transmission spectra were recorded at room temperature using a POLON type spectrometer and linear arrangement of source, a multichannel analyzer with 1024 channels (before folding), the absorber and a detector. A source of about 15 mCi activity ^57^Co in rhodium matrix (Cyclotron Co., Ltd, Obninsk) was used at room temperature. A gas proportional counter LND–45431was used as a gamma-ray detector. A 2-mm plastic filter was placed in the beam to absorb the 6 keV X-rays before they entered to the detector. The 2 keV escape peak and 14.4 keV gamma ray pulses were selected with a multichannel analyzer. The spectrometer was calibrated at room temperature with a 10 μm thick α-Fe foil. The line shapes were pure Lorentzian with the first and the sixth, the second and the fifth, the third and the fourth line widths values of *Γ*_1–6_ = 0.17 mm s^−1^, *Γ*_2–5_ = 0.15 mm s^−1^ and *Γ*_3–4_ = 0.15 mm s^−1^ for the α-Fe spectrum. The measured 1024 channel Mössbauer spectra were converted into 512 channels by consequent summation of two neighboring channels to increase the signal-to-noise ratio for the lowest-intensity spectra components. The numerical analysis of the Mössbauer spectra was performed with the use of the WMOSS program (Ion Prisecaru, “WMOSS4 Mössbauer Spectral Analysis Software” http://www.wmoss.org/, 2009–2016). The obtained spectra were fitted as a superposition of several doublets. The decomposition into doublets was performed by a Lorentzian function.

## Results and discussion

The chemical composition of the investigated olivine from Lanzarote is shown in Table 1. This composition leads to a mean global formula of olivine (Mg_0.896_Fe_0.104_)_2_SiO_4_ (without taking into account the minority elements Ni, Ca, Al, and Ca).

**Table 1:** The chemical composition of the investigated initial olivine sample

	Mg	Fe	Si	Ni	Ca	Al	Mn
wt%	25.43	6.78	17.60	0.34	0.16	0.56	0.11

**Fig. 2:**
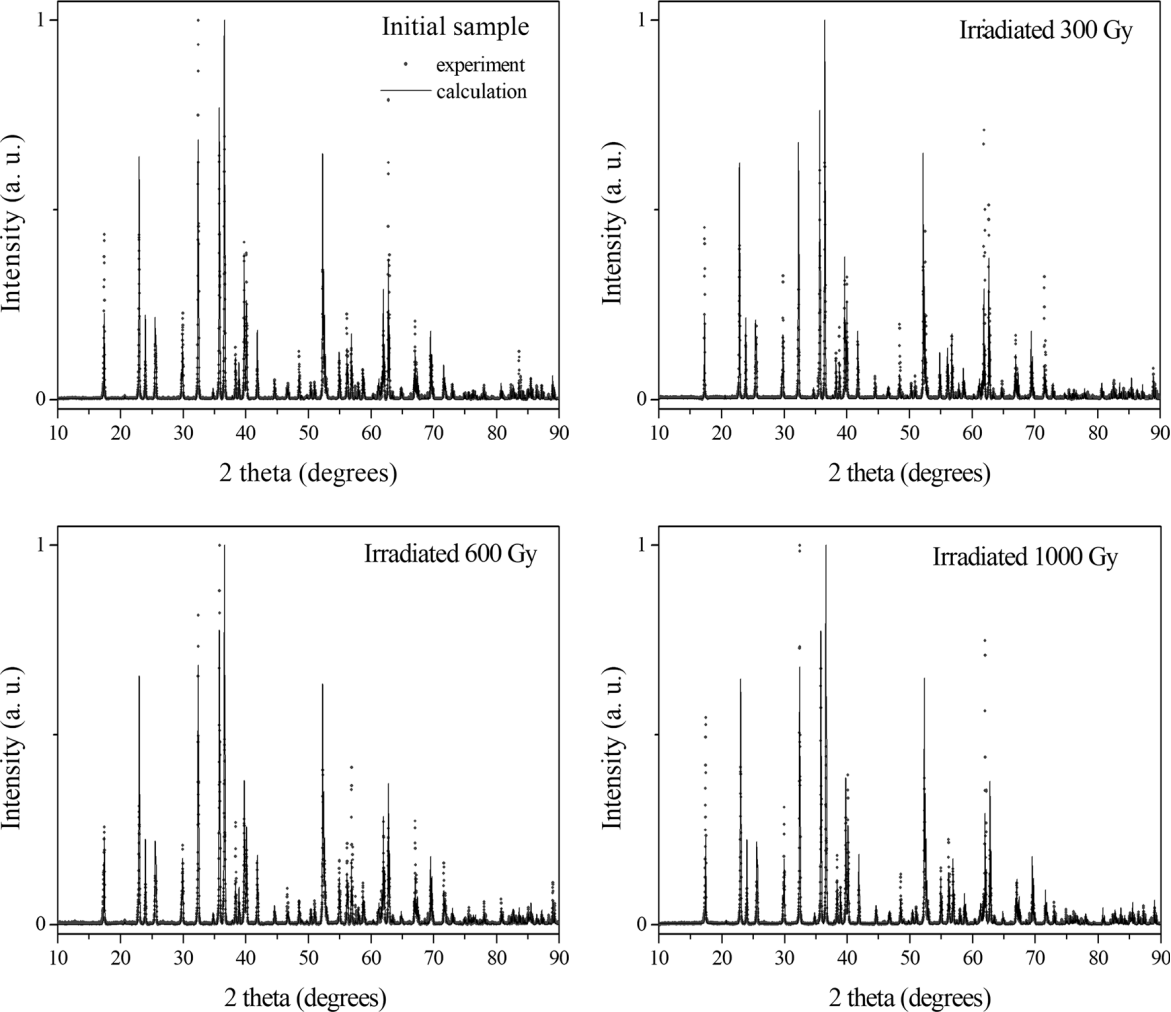
Diffractograms of initial and gamma irradiated olivine

Diffractograms of initial and gamma irradiated olivine are presented in Fig. 2. Obtained XRD patterns show the same main lines as those in the theoretical end experimental diffractograms of olivine presented in literature [11, 12]. The calculated chemical formula of the studied olivine (Mg_0.905_Fe_0.095_)_2_SiO_4_ is almost identical with those obtained from XRF calculation. Orthorhombic lattice parameters (space group *Pbnm*) determined by 266 positions reflections between 15° and 90° 2 theta (2Θ) are listed in Table 2. The calculated values are close to olivine parameters rich in magnesium [1, 13] and stay almost constant during the whole irradiation process. Consequently, the volume of unit cells of olivine after the absorbed doses stays almost constant. The main olivine phase is observed after absorbed dose from 300 to 1000 Gy. On the diffractograms (Fig. 2) no additional lines that may come from different crystal phases or superstructure were observed. However, significant modifications of some line intensities were observed, for example, lines (301) 2Θ = 32.40° (311) 2Θ = 35.78° (402) 2Θ = 52.59°, and (040) 2Θ = 62.00°. Such changes of intensity lines without changing lattice parameters can be a result of mutual twist of octahedrons (and tetrahedrons) under effect of gamma irradiation. Such rotation change distance between of oxygen, iron, magnesium and silica ions and can lead to deformation inside the unit cell. The second reason for changing intensity lines on the obtained diffractograms can be the increase of lattice vibrations influenced by gamma irradiation which supplies additional energy to the crystal. Both of these reasons can change the number of diffracting particles on the plane and therefore change the intensity line on XRD diffractograms.

**Table 2:** Lattice parameters and cell volumes obtained by Rietveld refinements of initial and irradiated olivine

	Mg_2_SiO_4_	Fe_2_SiO_4_	Initial olivine	Irradiated		
				300 Gy	600 Gy	1000 Gy
a (Å)	5.9817(5)	6.0902(5)	5.9942(1)	5.9908(2)	5.9932(2)	5.9933(2)
b (Å)	10.1978(8)	10.4805(8)	10.2261(3)	10.2212(4)	10.2249(4)	10.2270(3)
c (Å)	4.7553(3)	4.8215(5)	4.7641(1)	4.7604(2)	4.7633(2)	4.7631(2)
V (Å^3^)	290.07(6)	307.75(8)	292.02(6)	291.49(4)	291.89(4)	291.94(6)

Literature data for forsterite [11] and fayalite [12] are given for comparison

**Fig. 3:**
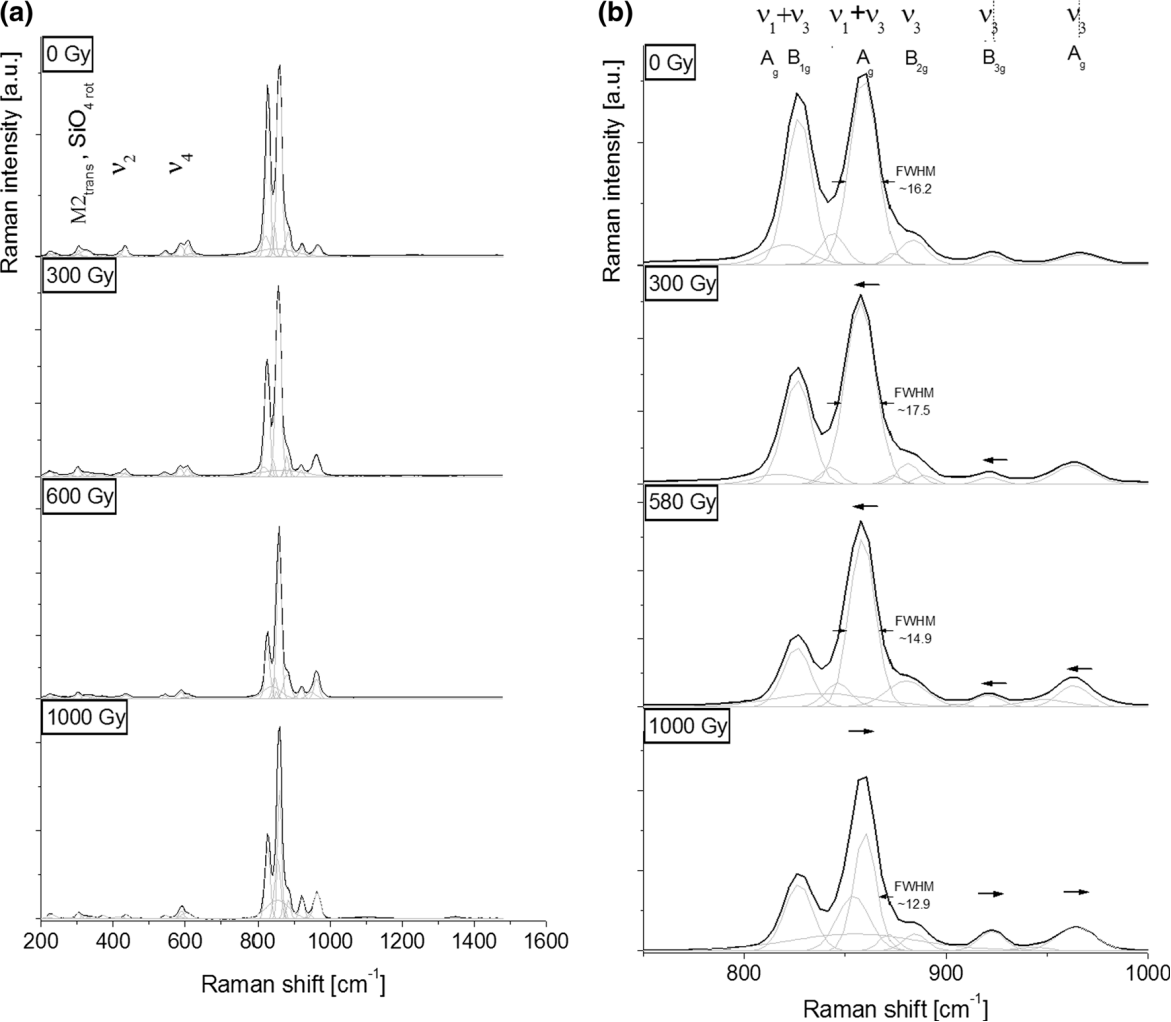
Raman spectra of an initial and gamma irradiated olivine in fingerprint 200–1600 cm^−1^ range (**a**) and magnified to better visualization of the characteristic olivine band doublet of 750–1000 cm^−1^ region (**b**). Long horizontal arrows indicate band shift while short arrows describe FWHM of the most intense band at 859 cm^−1^

Raman spectrum of magnesium olivine (= forsterite) is strongly determined by the crystal structure in which tetrahedral coordinated Si(IV) forms isolated SiO_4_ units in which each oxygen atom is shared with three octahedral M(II) (M(II) = Mg, Fe edges. This linkage introduces into the crystal field a distortion observable both within the octahedral and tetrahedral units. Olivine crystal structure is distinguishable through two crystallographically distinct M(II) sites, that is, a. smaller M1 (*C*_*i*_ symmetry) site and a larger M2 (*C*_*s*_ symmetry) site with a shorter average M1–O bond length (2.094 Å) relative to bond lengths (2.129 Å) observed within M2 octahedra [14, 15]. Octahedral units share six or three edges with neighbouring polyhedra, wherein only two or one with SiO_4_ tetrahedra, respectively. The presence of two different octahedral units strongly determines the character of SiO_4_ internal modes corresponding to the position full width at half maximum, or band asymmetry in relation to Raman data. One can note that the M1 site is very sensitive to an isomorphic substitution of magnesium through ferric ion. However, translation movements ascribed to M1 octahedral units are forbidden for Raman due to inversion symmetry and they have no special impact on the spectrum. In turn, translation movements of M2-O are strongly equipped with rotational ones of the SiO_4_ group resulting in silicon-oxide tetrahedra band position. The M2 site is very sensitive to isomorphic substitution Mg → Fe, usually providing extension of bonds in M2 octahedra with an increased concentration of ferric ion. Such effects are correlated with bond asymmetry within neighbouring tetrahedral or octahedral units and are evidenced due to mirror plane symmetry on the Raman spectrum. Isomorphic substitution in this position may also locally generate structural distortion within the M_2_SiO_4_ framework in the form of electron vacancy or point defects implying modification of FWHM value of silicon-oxide tetrahedra bands. Such structural implications are reflected on the Raman spectrum of typical olivine, especially when looking more closely at spectral ranges: (1) 700 ÷ 1100 cm^−1^ (2) 400 ÷ 700 cm^−1^, and (3) < 400 cm^−1^ (Fig. 3a). The bands of region (1) are the most prominent through the analysis of structural data of olivine-like phases and give an opportunity to distinguish between different types of olivine. Here, the high intensity doublet of bands cantered about 859 and 826 cm^−1^originates from the coupled stretching ν_1_ + ν_3_ mode activated within the silicate SiO_4_ group (Fig. 2b) [15]. The degree of such coupling is, in turn, sensitive to isomorphic substitution, especially to the size of the cations occupying the M1 and M2 sites, which determine the degree of distortion within the neighbouring polyhedra. As a result, distortion and coupling between ν_1_ and ν_3_ modes of the SiO_4_ tetrahedra is decreased as the differences between the M1 and M2 are reduced. These observations are strongly correlated with band asymmetry or position and they are most susceptible to structural changes within the crystal field of the olivine. In turn, four low intense bands localized at 966, 922, 884, and 874 cm^−1^ originate from ν_3_ vibration of the SiO_4_ group and different symmetry-type (B_3g_, B_2g_, B_1g_) [15, 16]. The other bands with relatively high value of FWHM are cantered in places that are not described on the basis of previous studies performed on similar olivine crystals. They may result from local disturbance of the crystal structure as a result of isomorphic substitution Mg → Fe, which implies formation of point defects related to the skew of silicon-oxygen tetrahedra from their ideal crystallographic position. Low intense bands of the region (2) are assigned to the deformational ν_2_ and ν_4_ mode of SiO_4_ group. In more detail, four bands centred at 607, 587, 544, and 433 cm^−1^ originate from ν_4_(B_1g_) + ν_2_(B_2g_) and ν_2_(B_1g_) + ν_2_(B_2g_) modes and are well correlated with typical Earth-origin olivine and meteoric olivine [17]. Like previously, additional bands with very low intensity are linked to the point defects appearing due to local modification in atom arrangement, especially within the SiO_4_ unit. A series of overlapping bands of the region (3), in turn, originate typically from the lattice mode with translation and rotation movement within silicon-oxygen tetrahedron equipped with the atomic movement within the M2 octahedral sites [14, 15, 17].

**Fig. 4:**
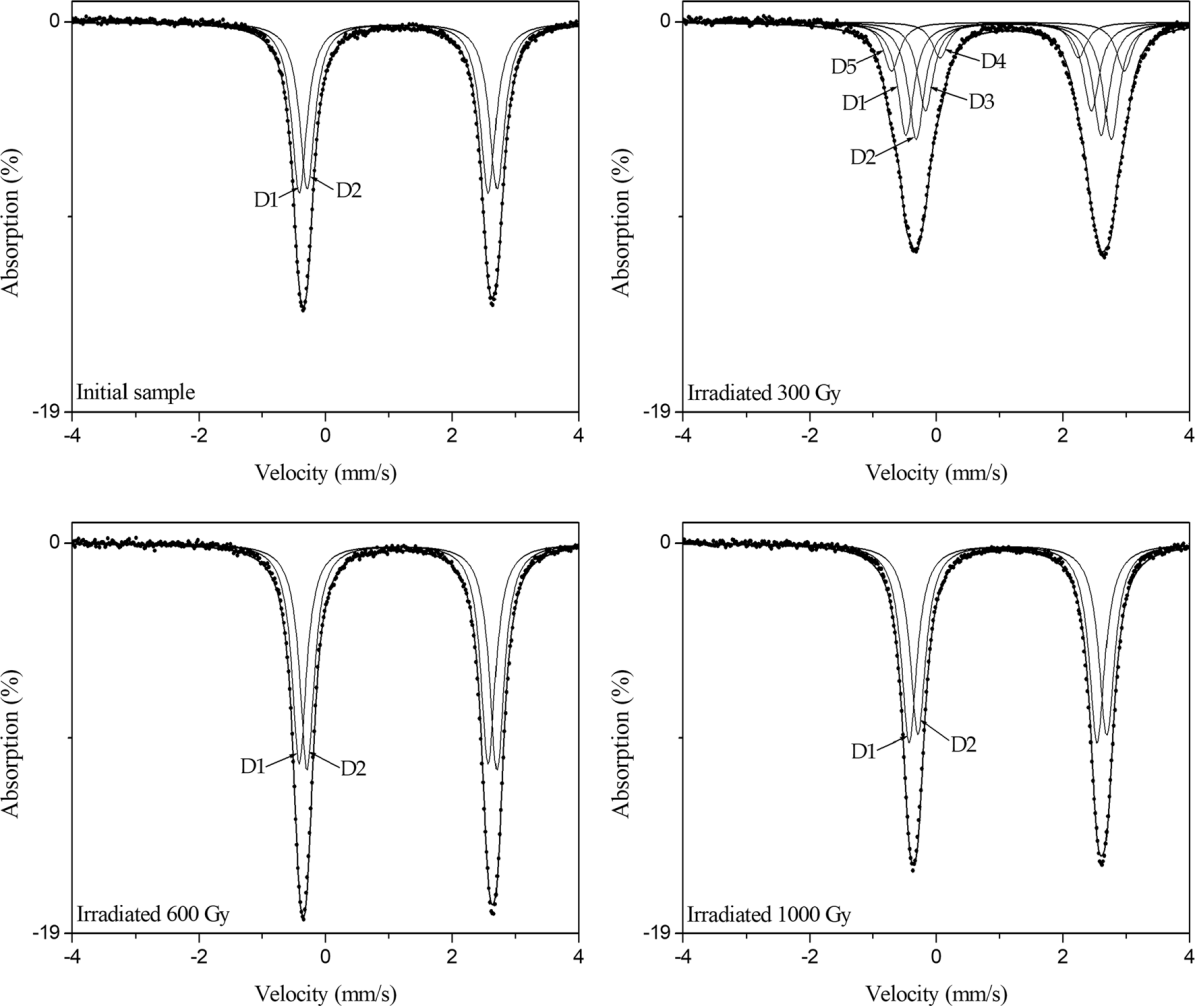
Room temperature Mössbauer spectra obtained for initial and gamma irradiated olivine. The fitted subspectra are presented on the investigated spectra

For Fe–bearing minerals the most suitable method for researching the valence and coordination of Fe ions is Mössbauer spectroscopy method [18–20]. What is most important, we consider an iron nuclide as a probe of local surrounding in crystal lattice. Changing hyperfine parameters brings information about changes in the sample [21–23]. Figure 4 shows room temperature Mössbauer spectra obtained for investigated olivine sample before and after absorbed doses (300, 600 and 1000 Gy). Table 3 sums up the hyperfine parameters obtained for all subspectra for the initial and gamma irradiated olivine. The Mössbauer spectrum of non-irradiated olivine contain two doublets that originate with Fe(II) ions in the octahedral positions of the olivine structure. The doublets with smaller quadrupole splitting (i.e. more distorted octahedron), can be assigned to Fe at M1, the other one with the larger quadrupole splitting to Fe at M2 [24]. After an absorbed dose of 300 Gy the Mössbauer spectrum changed drastically. On this spectrum five ferrous doublets are visible. Two of them have hyperfine parameters close to those observed in the initial sample. Abundance of these components decreases because additional tree doublets are present on the spectrum. The isomer shift of doublets D3, D4 and D5 are approximately the average of isomer shift doublets D1 and D2. However, they have completely different values of quadrupole splitting from small value 2.19 mm s^−1^ to huge 3.68 mm s^−1^. Changes of isomer shift values are connected with the change of local density of s-electron at the iron nucleus, and changes of quadrupole splitting are produced by the change of an asymmetric electronic charge distribution which are generated by changing local surrounding of Fe nuclide. Such modifications are the direct consequences of interaction of gamma rays with matter, which can lead to atomic displacement, namely vacancy-interstitial pairs of small separation, randomly distributed through the lattice. Gamma rays also produce ionization in the sample, electrons are ejected with energy comparable to the original γ-ray energy and can be brought far away from the initial place. Additionally, irradiation causes excitation of atoms in lattice, which is a similar effect to high temperature action, and that is why values of hyperfine parameters can be changed by second-order Doppler effect. After the absorbed dose of 600 and 1000 Gy the Mössbauer spectra did not change too much compared with the spectrum of the initial sample, which is reflected in the values of hyperfine parameters of their subspectra. It can indicate ordering of the crystal structure of olivine. It should be noticed that although the values of hyperfine parameters (except for 300 Gy) and abundance of Mössbauer subspectra were only slightly changed, the values of absorption were changed significantly. This signal indicates the change of the conditions for the occurrence of the Mössbauer effect which is strongly connected to the lattice and temperature. Therefore, the use of the gamma irradiation produces effects similar to the ones caused by high temperature. Applied small absorbed doses causes an increase in lattice vibrations, small deformation of both M1 and M2 octahedron and consequently runaway of defects.

**Table 3:** The Mössbauer hyperfine parameters of the initial and gamma irradiated olivine

Sample	Component	Is (mm s^−1^)	Qs (mm s^−1^)	G (mm s^−1^)	A (%)
Initial	D1	1.08	2.99	0.31	51
	D2	1.21	3.00		49
300 Gy	D1	1.07	3.09	0.32	28
	D2	1.23	3.08		29
	D3	1.16	2.19		9
	D4	1.15	2.62		22
	D5	1.13	3.68		12
600 Gy	D1	1.08	2.99	0.31	49
	D2	1.21	3.00		51
1000 Gy	D1	1.06	2.97	0.30	51
	D2	1.20	2.98		49

*Is* isomer shift, *Qs* quadrupole splitting, *G* full line width at half maximum, *A* area fraction of subspectra

## Conclusions

This review presents data on the influence of gamma irradiation (dose 300, 600, and 1000 Gy) on the crystal structure of olivine of chemical formula (Mg_0.905_Fe_0.095_)_2_SiO_4_ using X-ray diffraction, Raman spectroscopy, X-ray fluorescence method, and Mössbauer spectroscopy. Studies show that each of these methods gives interesting results but we can obtain clear explanation of the observed phenomena when results of one of the methods are confirmed and completed by results of another. The Mössbauer spectroscopy results indicated on the appearance of lattice vibrations and small deformation of octahedrons (simultaneous tetrahedrons) in olivine structure also confirms Raman measurements, which has structural modification correlated with bands shift towards a higher wave number. The uses of the absorbed dose lead to lattice vibrations, small deformations and runaway of defects, but all changes take place only in the volume of the unit cell of the olivine crystal, which can be acknowledged in an X-ray diffraction study.
